# *Galeata*: chronic migraine independently considered in a medieval headache classification

**DOI:** 10.1186/1129-2377-15-16

**Published:** 2014-03-21

**Authors:** Ángel Luís Guerrero-Peral, Virginia de Frutos González, María Isabel Pedraza-Hueso

**Affiliations:** 1 Neurology Department, Hospital Clínico Universitario, Avda Ramón y Cajal 3, 47005 Valladolid, Spain; 2 G. I. R. Speculum medicinae, University of Valladolid, Valladolid, Spain

**Keywords:** Chronic migraine, Constantine the African, Galeata, Headache classifications

## Abstract

**Background:**

Chronic migraine is a quite recent concept. However, there are descriptions suggestive of episodic migraine since the beginning of scientific medicine. We aim to review main headache classifications during Classical antiquity and compared them with that proposed in the 11th century by Constantine the African in his *Liber Pantegni*, one of the most influential texts in medieval medicine.

**Method:**

We have carried out a descriptive review of Henricum Petrum's Latin edition, year 1539.

**Results:**

Headache classifications proposed by Aretaeus of Cappadocia, Galen of Pergamun and Alexander of Tralles, all of them classifying headaches into three main types, considered an entity (called *Heterocrania* or *Hemicrania),* comparable to contemporary episodic migraine.

In ninth book of *Liber Pantegni*, headaches were also classified into three types and one of them, *Galeata*, consisted on a chronic pain of mild intensity with occasional superimposed exacerbations.

**Conclusion:**

In *Liber Pantegni* we have firstly identified, as a separate entity, a headache comparable to that we currently define as chronic migraine: *Galeata.*

## Background

Headache is as old as humanity. Given its prevalence and the disability to which it may lead to, headache has been immersed in the emotions and beliefs of all ancient civilizations. Therefore, from the beginning of medicine, authors have tried to understand and classify different types of headache [1,2].

The concept of chronic migraine was suggested in the descriptions by Mathew in 1982 [3,4], and in the diagnostic criteria of chronic daily headache proposed by Silberstein, including the transformed migraine [5]. The second edition of International Classification of Headache Disorders (ICHD-II) included Chronic Migraine among complications of migraine, and described it as a headache on 15 or more days per month for more than three months, in the absence of medication overuse, and fulfilling the criteria for migraine without aura of the same classification, ie, at least two characteristics among unilateral location, pulsating quality, moderate or severe pain intensity and aggravation by physical activity, and at least one symptom among nausea and/or vomiting, photophobia and phonophobia [6].

These criteria were found too restrictive and, shortly after ICHD-II publication, a subcommittee of the International Headache Society developed a revision of chronic migraine criteria in order to reflect in a better way the reality of clinical practice. Thus, it was considered in this broader concept that a patient with chronic migraine should present headache 15 or more days per month, and, among them, 8 or more with migrainous characteristics [7]. These new criteria were quickly accepted by the headache community, and were so included in the provisional third edition of International Classification of Headache Disorders (ICHD-III beta) [8]. The new classification considered chronic migraine as an independent type of migraine, instead of a complication, and permitted to diagnose in a same patient chronic migraine and medication-overuse headache.

We aim to review main headache classifications during Classical Antiquity and to compare them with a classification proposed in 11th century *Liber Pantegni*, one of the most influential texts in medieval medicine. We have carried out a descriptive review of Henricum Petrum's Latin edition of *Liber Pantegni*, year 1539 [9].

## Methods

### Headache classifications during classical antiquity

Plinius the Elder (23–79 AD) proposed the first headache classification considering different pain sites (temples, occipital, holocranial) [2].

Aretaeus of Cappadocia (30–90 AD) [1,10,11] classified headaches in three main types: *Cephalalgia* is a pain related to a systemic disturbance, usually of mild intensity and short duration, tough it can be dangerous if associated with fever, chills, or hypotonia. *Cephalea* is a chronic and more severe headache, sometimes remitting and commonly refractary to therapy. Finally, *Heterocrania* consists on a paroxysmal headache located on one side of the head, with changing location and intensity, and usually accompanied by nausea, bilious vomiting, sweating, dizzyness, photophobia or changes in perception of fragances. *Heterocrania*, so, is comparable to current migraine, and can become chronic, of mild intensity and accompanying awkwardness, light-headedness, anxiety and boredom. Unfortunately, remedies are scarce and, except purgatives and bloodletting in *heterocrania,* not specific for different types of headache.

Galen of Pergamum (129–199 AD) [2,12] was the organizer of classical medical knowledge, with a large influence in medieval medicine. In his headache classification he considered also three main types: *Cephalaia* is similar to *Cephalalgia* of Aretaeus. *Hemicrania*, comparable to Aretaeus *Heterocrania,* is due to an excesive amount of yellow bite, with a throbbing pain component due to arterial pulsation [1,13]. Finally, *Cephalea* is a chronic and persistent pain with occasional superimposed paroxysms of greater intensity accompanying photo and photophobia.

Byzantine medicine followed galenic classification of headaches into *cephalaia, cephalea* and *hemicrania*[1,14-18]. Alexander of Tralles (525–605 AD) [16], dedicated the book I of *Medici libri duodecim* to head diseases [19]. He slightly modified Galen classification. He considered *Cephalalgia*, pain secondary and brief, with worse prognosis only if posttraumatic, *Cephalea*, chronic pain with pain-free intervals and arising from unimportant causes. Finally, *Hemicrania,* due to an excess of yellow bile and comparable, as Areteo’s *heterocrania* and Galen’s *hemicrania*, to contemporary episodic migraine.

### Constantine the African and his time

Whilst Byzantium preserved Greek-Roman medical science, in Western Europe impoverished medical knowledge sheltered in the monasteries [20,21]. Slow renaissance of medieval Western medicine had a determinant milestone in Salernitan Medical School. Salerno, located in southern Italy, was a crossroads of civilizations (Normans, Arabs, Byzantines) sheltered by Benedictine monastery of Monte Cassino. Here, inside mythological references, a medical school was founded sometime between nine and tenth century, thanks to collaboration between Greek, Latin, Jewish and Arab physicians. Salerno was a School, from the beginning, primarily secular and restricted to medical teaching [20,22-25]. In Salerno teaching took place initially with the support of the oral tradition, but teachers quickly realized the needing for texts, achieving some Byzantine or Latin books.

Some centuries earlier, Greek-Roman classical texts had migrated to the east with the Nestorian Christian heretics, and translated, first to syriac language, and then into arabic. Authors as Rhazes (864–935 AD) [2,26] or Avicenna (980–1037 AD) [1,2] represents Islamic medieval medicine, one of the most brilliant periods in the history of medicine [27].

There is a key moment in history of medicine, in which Arabic medicine contributed to the renaissence of Western medieval medicine: the arrival of Constantine the African to the School of Salerno [22,23,28,29].

Constantine the African (1010–1087) is one of the most attractive figures in history of medicine but his biographies are imbued with legendary items. Born in Cartaghe, probably under the Arab rule, he studied medicine in Baghdad and extensively travelled through Syria, Egypt, Ethiopia and India, acquiring many Arabic medical texts. He returned to Cartaghe where he practiced medicine, tough he must flee accused of practicing magic. He then looked for refuge in Salerno when arrived in a vague date among 1065 and 1077. He lived in Monte Cassino and taught medicine at the medical school of Salerno. Abbot Desiderius and Alfano encouraged him to translate his Arab medical texts into Latin. These translations led to the most brilliant period of the School of Salerno and reintroduced Greek-Roman medical knowledge in Medieval Western Europe [30-33].

During these years Constantine translated from Arabic into Latin books of authors as Ibn Al-Gazzar (*Viaticum*), Johannitius (*Isagoge*), Hippocrates (*Aphorisma, Prognostica*), Galen (*Tegni, Megategni*) Philareto (*De pulsibus*), Rhazes (*Liber divisionum, Liber experimentorum*) or Isaac Iudeus (*Liber dietorum, Liber urinarium, Liber febrium*). In these translations, though respecting main doctrinal concepts and general structure of the books, he maked multiple contributions which are often difficult to differentiate from the original texts; he also removed references to the original authors passing the books as their own [32,34,35]. The debate about the alleged plagiarism in Constantine’s texts opened in the twelfth century and remains alive. In discharge of Constantine, it shoud be pointed that he transmitted to Latin new medical concepts unknown in that language, and that he contributed with original ideas in all his books. However, it must be critizised that he did not mention the authors of the texts he translated, except for Isaac Iudeus [15].

## Results

### Liber Pantegni

It is one of the most important and influencing books translated by Constantine the African [36]. A text of a Persian physician named Ali ibn Abbas al-Majusi (Haly Abbas in Latin) (930–994 AD) called *Kamil as-Sina'a at-tibbiya* (The Complete book of the medical art), or *Kitab al-Maliki* (Royal Book) was Constantine first translation titled as *Pantegni*. It rapidly became the leading textbook of medicine at the first European universities and medical schools.

Little is known about Haly Abbas except for his birth in Ahwaz, near Gundishapur, in a family that professed Zoroastrianism. We also know that he dedicated his book to a prince named Adud al-Dawla, probably from Buyida Dynasty in Baghdad, whom he served as a physician [37-40]. Constantine the African translated this book in 1087 without mentioning Haly Abbas as the author. To complete the history of the text, a new Latin translation was done by Stephen of Antioch in 1127, entitled Liber Regius [41]. *Liber Pantegni* contains 10 books (1. Generalities about medicine, 2. Simple members description, 3. Compound members description, 4. Sensory organs functions, 5. The *galenic sex res non naturales*, 6. Sensory organs diseases, 7. Pulse, digestion and urine pathologies, 8. Skin diseases, 9. Therapeutic teatrise *a capite ad calcem*, and 10. Natural history of some diseases). Each book is divided into multiple chapters.

*Pantegni* and its Arabic model, *Kitab*, are both divided into two sections of ten books in each one, called *Theorica* and *Practica*. The ten books of *Theorica Pantegni* correspond to the first ten books of *Kitab*, but it seems that Constantine left *Practica* unfinished. When mentioning headache classification in *Liber Pantegni*, we will cite chapters and pages according to the aforementioned Petrum Henricum edition of 1539 [9].

A whole chapter of ninth book of *Liber Pantegni* is dedicated to headaches. In headache classification there are also three different types of headache. *Cephalea* is defined as a holocraneal pain due either to systemic diseases or trauma. Environmental factors could favour it as cold winters *(Book V, chap. V, p. 104)*, or some foods as onion *(Book V, chap. XVII, p. 124)*, milk, warm honey with nuts *(Book V, chap. XXVI, p. 130)*, and wines, especially red ones *(Book V, chap. XXVIII, pp. 134–135)*; this type of headache is comparable to previous *cephalalgia* or *cephalea. Hemicrania*, in *Liber Pantegni* classification, is comparable to that described by Galen and it consists on a hemicranial pain caused by meningeal disturbances related to bad humours or slow digestions. Sometimes it associates loss of vision *(Book IX, chap. III, p. 243).*

Galeata is, in our opinion, the most original part of the classification. It consist on a chronic pain, commonly of mild intensity, with occasional superimposed exacerbations triggered by noise, heat vision, smells or wine intake. As Constantine describes: “Headache will be prolonged and difficult to cure, slightly painful until it reaches more suffering, so patient cannot bear to hear a voice or a slightly noisy conversation, nor movements or lights. Patient prefers to remain silent and in darkness due to the great pain he feels”^a^.

Sometimes, pain may radiate to eyes. Therapy of *Galeata* is considered ineffective *(Book IX, chap. III, p. 243). Galeata* takes the place of *cephalea* in previous classifications. Its characteristics are similar to Galen’s *cephalea*, although the different name helps to distinguish this entity from Tralles or Aretaeus definition.

## Discussion

Table 1 compares *Liber Pantegni* with most important headache classifications of classical antiquity.

**Table 1:** **Comparison among main headache classifications in Classical antiquity and *Liber Pantegni***

**AUTHOR (Reference)**	***Term***		
	**Description**		
**ARETAEUS**[10,11]	***Cephalalgia***	***Cephalea***	***Heterocrania***
	Mild intensity and short duration	Chronic and severe headache	Paroxysmal headache
	Secondary to a systemic problem	Sometimes refractory to therapy	Hemicranial location
**GALEN**[1]	***Cephalaia***	***Cephalea***	***Hemicrania***
	Mild intensity and short duration	Chronic and persistent pain	Paroxysmal throbbing headache
	Secondary to a systemic problem	Superimposed paroxysms	Hemicranial location
**TRALLES**[19]	***Cephalalgia***	***Cephalea***	***Hemicrania***
	Pain brief and secondary	Chronic pain. Pain-free intervals	Paroxysmal headache
	Worse prognosis if posttraumatic	Unimportant causes	Hemicranial location
**LIBER PANTEGNI**[9]	***Cephalea***	***Galeata***	***Hemicrania***
	Holocranial pain	Chronic mild pain	Paroxysmal headache
	Secondary to systemic diseases or trauma	Superimpose exacerbations with photo-phonophobia and aggravation by physical activity	Hemicranial location

## Conclusion

There are interesting proposals of Headache classifications in Classical Antiquity. Episodic migraine was well defined from the beginning of these classifications and after Galen, was named *Hemicrania.*

According to our review of Liber Pantegni, this book, one of the most influential ones in Western medieval medicine, contains the first description of a headache comparable to what we consider nowadays a chronic migraine, and independently considered in a headache classification. Its name: *Galeata.*

## Endnotes

^a^*“cephalea erit diuturna ad sanandum dura, parum nociva, donec in maius nocumentum veniat, ut nullam vocem tangibilem sustinere valeat, nec sermonem aliquatulum clamoris habentem, nec motum, vel splendorem aliquem. Sed maxime amat ut in quiete, et obscuritate maneat propter magnitudinem doloris quem sentit”*.

## Competing interest

The authors declare that they have no competing interest.

## Author’s contribution

F-G V. reviewed and translated latin text. G-P AL and P-H MI drafted the manuscript. All authors read and approved the final manuscript.
